# Potential Usefulness of FDG PET/CT in Patients with Sepsis of Unknown Origin

**DOI:** 10.1371/journal.pone.0066132

**Published:** 2013-06-11

**Authors:** Jing-Ren Tseng, Ke-Yuan Chen, Ming-Hsun Lee, Ching-Tai Huang, Ying-Hao Wen, Tzu-Chen Yen

**Affiliations:** 1 Nuclear Medicine and Molecular Imaging Center, Chang Gung Memorial Hospital at Linkou, Taoyuan, Taiwan, R.O.C.; 2 Division of Infectious Diseases, Department of Internal Medicine, Chang Gung Memorial Hospital at Linkou, Taoyuan, Taiwan, R.O.C.; 3 Department of Pathology, Chang Gung Memorial Hospital at Linkou, Taoyuan, Taiwan, R.O.C.; The University of Chicago, United States of America

## Abstract

**Purpose:**

The role of FDG PET in the evaluation of patients with sepsis of unknown origin remains unclear. We sought to assess the value of FDG PET/CT in patients with sepsis of unknown cause and to define its priority in this group of subjects.

**Methods:**

A total of 53 patients with sepsis of unknown origin underwent FDG PET/CT within two weeks of diagnosis. All of the patients were followed up for at least 3 months after discharge to determine the clinical outcomes. The impact of FDG PET/CT was assessed according to the number of cases who had their treatment modified on the basis of the imaging results. Logistic regression analysis was used to identify the independent predictors of positive FDG PET/CT findings.

**Results:**

Of the 53 study patients, 35 (66%) had positive FDG PET/CT findings, and 13 (25%) had their treatment modified on the basis of the imaging results. Logistic regression analysis identified normal serum aspartate aminotransferase (odds ratio [OR]  = 6.134; 95% confidence interval [CI]  = 1.443–26.076, *P* = 0.014) and increased serum alkaline phosphatase levels (OR = 5.813; 95% CI = 1.386–24.376, *P* = 0.016) at diagnosis as independent predictors of positive FDG PET/CT findings. A scoring system using these two covariates was developed, which defined three distinct priority groups for FDG PET/CT imaging.

**Conclusion:**

Our findings suggest that FDG PET/CT may be clinically useful for the detection of occult foci of infection in patients with sepsis of unknown origin.

## Introduction

The identification of occult sources of infection is of paramount importance to improve the outcomes of patients with sepsis [1]. In recent years, several radiopharmaceuticals have been used for the detection of infectious foci, including autologous white blood cells labeled with Technetium-99m (Tc-99m) or Indium-111 (In-111), Tc-99m-labeled bisphosphonates, and Gallium-67 (Ga-67) citrate [2]. Unfortunately, these compounds have inherent shortcomings, including being inconvenient and time-consuming (autologous white blood cells labeling), having low specificity despite a high sensitivity (Tc-99m labeled bisphosphonates), and producing suboptimal image quality (Ga-67 citrate scan). Moreover, the poor spatial resolution of single-photon emission computed tomography (SPECT) has limited the wide clinical use of these radiopharmaceuticals.

Compared with conventional imaging modalities, fluorodeoxyglucose positron emission tomography/computed tomography (FDG PET/CT) offers important advantages as it can provide both metabolic and structural/anatomical information of a lesion. In addition, whole-body FDG PET/CT scans require significantly less time than whole-body magnetic resonance imaging (MRI) [3] and are not contraindicated in patients with renal failure or implanted metal devices. Previous studies have shown that FDG PET/CT is superior to conventional imaging modalities in several infectious and inflammatory disorders, including osteomyelitis, infected vascular grafts, metastatic infectious disease, vasculitis, sarcoidosis, inflammatory bowel disease, and fever of unknown origin [2], [3]. However, the role of FDG PET/CT in the evaluation of patients with sepsis of unknown origin is still unclear. In this retrospective study, we sought to assess the value of FDG PET/CT in subjects with sepsis of unknown origin and to define its priority in this group of patients. The impact of FDG PET/CT was assessed according to the number of cases with treatment modifications based on the imaging results.

## Methods

### Ethics statements

This retrospective observational study complied with the tenets of the Declaration of Helsinki and was approved by the Institutional Review Board of the Chang Gung Memorial Hospital (CGMH) at Linkou (Number: 101-4533B). The requirement of informed consent was waived. However, informed consent was required upon admission for all of the medical procedures. All of the data were securely protected (by delinking identifying information from the main data sets), made available only to investigators, and analyzed anonymously.

### Patients and clinical management

Between October 1, 2011 and September 30, 2012, we identified a total of 53 patients (median age: 68 years, age range: 38–97 years) who were admitted to the CGMH at Linkou because of sepsis of unknown origin and who underwent FDG PET/CT within two weeks of diagnosis. All of the patients received an extensive evaluation before FDG PET/CT imaging, including physical examination, blood tests, chest radiography, abdominal sonogram, CT scanning, and microbiological analysis. When the results were inconclusive and in the presence of clinical deterioration, FDG PET/CT was performed at the discretion of the attending physicians to identify occult foci of infection. We excluded patients with serum glucose levels above 200 mg/dL before the FDG injection and those who underwent FDG PET/CT after two weeks of the first positive blood culture. All of the patients were followed up for at least 3 months after discharge to determine the clinical outcomes.

Any condition associated with an increased risk of metastatic infectious foci (e.g., community-acquired infections, fever that persisted despite ≥72 hours of therapy, treatment started ≥48 hours after the onset of symptoms, positive blood cultures despite ≥48 hours of therapy, and the presence of prosthetic devices) [4]–[6] was carefully evaluated. All of the patients with bacteremia underwent empirical antibiotic treatment even though the focus of infection had not been yet identified.

### Definitions

All of the study participants had bacteremia and clinical evidence of the systemic inflammatory response syndrome (SIRS). SIRS was diagnosed according to the criteria of the American College of Chest Physicians and the Society of Critical Care Medicine [7]. Sepsis was defined as the clinical signs describing SIRS together with suspected or definitive evidence of infection [8]. Septic shock was defined as sepsis-induced hypotension (systolic blood pressure <90 mmHg or a reduction of 40 mmHg from baseline) in patients with signs of organ dysfunction despite adequate fluid resuscitation.

The baseline characteristics of the study patients were collected from their medical records. Infections that become clinically evident within 48 hours of hospitalization were considered as community-acquired. Chronic kidney disease and end-stage renal failure were diagnosed according to the National Kidney Foundation's Kidney Disease Outcomes Quality Initiative (K/DOQI) classification [9]. The diagnosis of liver cirrhosis was made by an expert gastroenterologist based on histological findings [10], imaging results, clinical data, and laboratory tests. A diagnosis of cancer included either solid-organ neoplasms or hematological malignancies. Blood culture results of hospitalized patients were retrieved using the computer-assisted microbiology reporting of our Clinical Microbiology Laboratory. The following definitions were used for laboratory testing: leukocytosis, white blood cell count >11,000/mm^3^; leukopenia, white blood count <3900/mm^3^; anemia, hemoglobin ≤12.0 g/dL; and thrombocytopenia, platelet count ≤150,000/µL. A red blood cell distribution width (RDW) >14.5% was considered as increased. A serum aspartate aminotransferase (AST) level <37 U/L was considered as normal, whereas an increased serum alkaline phosphatase (Alk-P) level was defined as >95 U/L.

### PET/CT acquisition, processing, and interpretation

All of the FDG PET/CT scans in this study were performed using a Biograph mCT PET/CT System (Global Siemens Healthcare, Erlangen, Germany) 50 min after the intravenous administration of ^18^F-FDG. The injected dose of ^18^F-FDG ranged from 370 to 444 MBq (10 to 12 mCi). Patients were asked to fast for 6 hours before examination. Insulin-dependent diabetic patients were scheduled to undergo their studies at noon and instructed to inject the adequate amount of insulin before the start of the fasting period. All of the subjects were scanned from head to mid thigh, with the exception of patients with lower limbs infections or peripheral arterial disease who underwent whole-body scans. CT data were used for both attenuation correction and fusion with attenuation-corrected PET images. All of the images (PET, CT, and PET/CT) were displayed in axial, coronal, and sagittal views. Moreover, PET data were displayed in a rotating maximum-intensity projection. All of the images were interpreted by a team of two nuclear medicine physicians and one radiologist. In cases of questionable findings, the decision was made by consensus of at least two observers. All of the readers were blinded to the patient's clinical history, blood tests, and previous imaging findings. Any abnormal uptake found to be greater than that of background uptake was considered as positive. We did not calculate the sensitivity and specificity of FDG PET/CT findings because of the lack of a gold standard in this patient group and the physiological uptake in the cerebral cortex and the urinary tract (which can make urinary tract and central nervous system infections difficult to detect). In consensus with the infectious disease physicians, abnormal PET/CT findings were defined as true-positive if they were confirmed by the clinical presentation or the complementary imaging workup. In contrast, normal FDG PET/CT results were considered as true-negative in the absence of localized foci and no recurrence of infection at follow-up.

### Statistical methods

Follow-up visits were continued until December 31, 2012. The descriptive statistics were summarized using frequencies, percentages, means, medians, ranges, and standard deviations (SD). The Kolmogorov-Smirnov test was used to evaluate deviations from a normal distribution for the continuous variables. We used independent Student's *t* tests and Mann-Whitney *U* tests to compare normally distributed and skewed continuous variables, respectively. The distribution of the categorical variables was analyzed using the χ^2^ test or the Fisher's exact test. We performed univariate and multivariate logistic regression analyses to identify the variables associated with positive PET/CT findings. All of the variables identified in univariate analysis were entered into a forward multivariate logistic regression model. The results of logistic regression were expressed as odds ratios (OR) and 95% confidence intervals (CI). Statistical calculations were performed using the SPSS software (version 16.0; SPSS Inc., Chicago, IL, USA). Two-tailed *P* values <0.05 were considered statistically significant.

## Results

### Microbiological findings

Of the 53 study patients, 31 (58%) had gram-positive bacteremia, 19 (36%) had gram-negative bacteremia, and 3 (6%) had multiple bacterial species isolated from blood cultures (i.e., polymicrobial bacteremia). The most common gram-positive pathogen was *Staphylococcus aureus*, which was isolated from 26 of 31 patients. The most common gram-negative pathogen was *Klebsiella pneumoniae*, which was isolated from 10 of 19 patients. For three patients with polymicrobial bacteremia, one had blood cultures positive for *Citrobacter koseri* (formerly called *Citrobacter diversus*), *Enterococcus faecalis*, and *Escherichia coli*; another had blood cultures positive for group B *Streptococcus* and *Proteus mirabilis*; and the other had blood cultures positive for *E. coli* and *Acinetobacter baumannii*. The prevalence of positive FDG PET/CT findings did not differ significantly among patients with blood cultures positive for gram-positive, gram-negative, or multiple bacterial species (*P* = 0.741).

### Clinical course and modification of treatment because of PET findings

**Figure 1:**
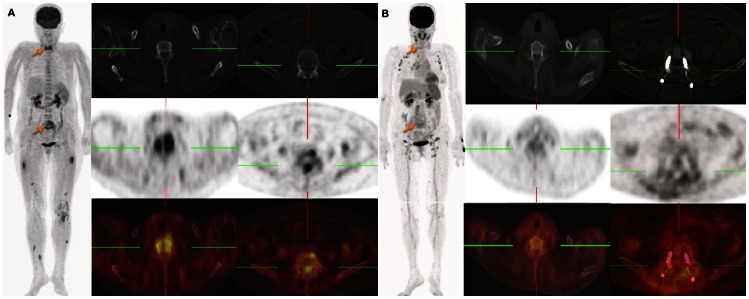
A 60 year-old male admitted with a diagnosis of *Staphylococcus aureus* sepsis of unknown origin. (A) The maximum intensity projection of PET and the transaxial views of CT, PET, and fused PET/CT images revealed an increased FDG uptake at C6 and L5-S1 in the spine (arrow). Subsequent MRI imaging confirmed the diagnosis of infectious spondylitis. Several metastatic foci were also bilaterally evident in the thighs and calves. (B) The patient underwent laminectomy at L4-S1 and interbody fusion. The pathological results confirmed the presence of necrotic bone and fibrous tissue to form an abscess. The results of follow-up imaging at 6 weeks showed the resolution of spine osteomyelitis (arrow). FDG PET/CT imaging demonstrated soft tissue inflammation in the postsacral region, as well as the presence of axillary and inguinal lymphadenopathy due to reactive hyperplasia.

A total of 35 (66%) patients had positive PET findings. Thirteen (25%) of the 53 study participants had their treatment modified because of PET results (Table 1 and Figure 1). Specifically, four patients received an additional drainage; of them, three were treated by CT-guided drainage and one underwent a percutaneous transhepatic cholangiography with biliary drainage. All of these patients were successfully cured. Nine patients were treated with surgery because of PET findings. Of them, six subjects were successfully cured, whereas three died. The presence of liver cirrhosis was the only variable significantly associated with the likelihood of negative PET data (*P* = 0.005; Table 2).

**Table 1:** Clinical characteristics of the study patients who had their treatment modification based on FDG PET/CT findings (n = 13).

No.	Age/Sex	Clinical history	Type of sepsis	Blood culture results	FDG PET/CT findings	Treatment modality	Clinical outcome	Days in hospital
1	84/M	LC	Nosocomial	*Citrobacter diversus, Enterococcus faecalis, Escherichia coli*	Biliary tract infection	PTCD	Cured	83
2	40/M	LC	Nosocomial	*Staphylococcus aureus*	Spine infectious spondylitis (L4-5)	Surgery	Cured	93
3	78/M	-	Community-acquired	*S. aureus*	Spine infectious spondylitis (T9-L4), psoas muscle abscess, pleural empyema	Surgery	Expired	60
4	52/F	-	Nosocomial	*Enterococcus faecium*	Intramuscular abscess of the adductor muscle of the thigh	CT-guided drainage	Cured	157
5	86/M	DM,HTN,COPD	Nosocomial	*Enterobacter cloacae*	Spine infectious spondylitis (T8-9)	Surgery	Expired	79
6	64/M	DM,HTN,CAD	Community-acquired	*S. aureus*	Spine infectious spondylitis (C5-6)	Surgery	Cured	50
7	60/F	-	Community-acquired	*S. aureus*	Spine infectious spondylitis (L5-S1)	Surgery	Cured	50
8	60/M	DM, HTN, LC	Community-acquired	*S. aureus*	Spine infectious spondylitis (T8-9)	Surgery	Expired	136
9	48/M	-	Community-acquired	*S. aureus*	Septic arthritis of the hip joint, iliac fossa abscess	Surgery	Cured	32
10	57/F	-	Community-acquired	*S. aureus*	Psoas muscle abscess	CT-guided drainage	Cured	29
11	60/M	DM,CKD	Community-acquired	*S. aureus*	Spine infectious spondylitis (C6-7, L5-S1)	Surgery	Cured	84
12	59/M	HTN,ESRD	Community-acquired	*S. aureus*	Psoas muscle abscess	CT-guided drainage	Cured	54
13	64/F	CAD,ESRD	Community-acquired	*S. aureus*	Spine infectious spondylitis (L3-4)	Surgery	Cured	64

M, male; F, female; LC, liver cirrhosis; DM, diabetes mellitus; HTN, hypertension; CAD, coronary artery disease; COPD, chronic obstructive pulmonary disease; CKD, chronic kidney disease; ESRD, end-stage renal disease; PTCD, percutaneous transhepatic cholangiography and biliary drainage.

**Table 2:** Baseline characteristics of patients with sepsis of unknown origin stratified according to the FGD PET/CT findings (n = 53).

Variable	Negative FDG PET/CT findings (n = 18)	Positive FDG PET/CT findings (n = 35)	*P*
Males, n (%)	10 (56)	22 (63)	0.607
Age, years	66.94±17.17	67.80±13.68	0.856
Older age (>65 years), n (%)	11 (61)	17 (49)	0.386
Community-acquired infection, n (%)	15 (83)	22 (63)	0.124
Diabetes mellitus, n (%)	10 (56)	13 (37)	0.200
Hypertension, n (%)	10 (56)	17 (49)	0.630
Cerebrovascular accident, n (%)	3 (17)	8 (23)	0.599
Coronary artery disease, n (%)	1 (6)	5 (14)	0.651
Chronic kidney disease, n (%)	3 (17)	3 (9)	0.397
Liver cirrhosis, n (%)	9 (50)	5 (14)	0.005*
Malignancy, n (%)	4 (22)	5 (14)	0.466
Chronic obstructive pulmonary disease, n (%)	1 (6)	2 (6)	1.000
End-stage renal disease, n (%)	4 (22)	4 (11)	0.299

*indicates statistically significant results.

We tested the association of the following variables with the presence of positive PET results: leukocytosis (presence *vs.* absence), leukopenia (presence *vs.* absence), anemia (presence *vs.* absence), increased RDW (presence *vs.* absence), thrombocytopenia (presence *vs.* absence), gram-positive bacteremia (presence *vs.* absence), normal AST (presence *vs.* absence), and elevated Alk-P (presence *vs.* absence). Normal AST and elevated Alk-P were significantly associated with the presence of positive PET findings in both univariate and multivariate analysis (Table 3). Of the 14 patients with liver cirrhosis, 9 had negative PET scans and 9 had increased AST levels. Nineteen patients had infections involving the musculoskeletal system (Table 4). All of them had positive PET scans and 16 had increased Alk-P levels. Twelve of these 19 patients had their treatment modification based on FDG PET/CT findings, and 9 survived without evidence of disease, whereas three died at the time of analysis (Table 1).

**Table 3:** Univariate and multivariate logistic regression analysis: predictive value of laboratory findings for positive FDG PET/CT findings in patients with sepsis of unknown origin (n = 53).

Variable (presence or not, n)	Univariate analysis		Multivariate analysis	
	OR (95% CI)	*P*	OR (95% CI)	*P*
Leukocytosis (Yes, 21)	1.500 (0.458–4.915)	0.503		
Leukopenia (Yes, 9)	0.583 (0.135–2.511)	0.469		
Anemia (Yes, 40)	2.000 (0.554–7.216)	0.290		
Increased red blood cell distribution width (Yes, 31)	1.200 (0.380–3.788)	0.756		
Thrombocytopenia (Yes, 24)	0.376 (0.117–1.211)	0.101		
Gram-positive bacteremia (Yes, 34)	1.220 (0.376–3.957)	0.741		
Normal AST (Yes, 34)	3.611 (1.088–11.984)	0.036*	6.134 (1.443–26.076)	0.014*
Increased Alk-P (Yes, 31)	3.429 (1.047–11.228)	0.042*	5.813 (1.386–24.376)	0.016*

OR, odds ratio; CI, confidence interval; AST, aspartate aminotransferase; Alk-P, alkaline phosphatase.

*indicates statistically significant results.

**Table 4:** Major septic foci identified in the 35 patients with positive FDG PET/CT findings.

System	Lesion	n (%)
Musculoskeletal system	Infectious spondylitis	13 (37)
	Septic arthritis	2 (6)
	Psoas muscle abscess	2 (6)
	Pyogenic myositis	1 (3)
	Infectious hematoma	1 (3)
Respiratory system	Pneumonia	8 (23)
	Septic emboli	3 (9)
	Lung empyema	1 (3)
Gastrointestinal system	Liver abscess	1 (3)
	Biliary tract infection	1 (3)
Genitourinary system	Lobar nephronia	1 (3)
Cardiovascular system	Infective endocarditis	1 (3)

### Priority of using FDG PET/CT in patients with sepsis of unknown origin

A scoring system using the two variables significantly associated with the presence of positive PET findings (normal AST and elevated Alk-P) was developed to define the priority of using FDG PET/CT in patients with sepsis of unknown origin. The score was constructed by summing up the two significant covariates, as follows: 0 for increased AST and 1 for normal AST; 0 for normal Alk-P and 1 for increased Alk-P. The scoring system defined three distinct priority groups for FDG PET/CT imaging: score 0, score 1, and score 2. The probability of a positive impact of FDG PET/CT decreased in a stepwise fashion from patients with a score of 2 (6/18, 33%), through those with a score of 1 (6/27, 22%), to patients with a score of 0 (1/8, 13%; Table 5). Among patients with a score of 2, 89% (16/18) had a positive FDG PET/CT scan, and six subjects had their treatment modified because of FDG PET/CT findings. Of them, five subjects survived and one died. In patients with a score of 1, 63% (17/27) had positive FDG PET/CT results, and six had their treatment modified. Of them, four survived and two died. In patients with a score of 0, 25% (2/8) had positive FDG PET/CT results. One patient had a treatment modification and was alive without disease.

**Table 5:** Triage scoring system defining three distinct priority groups for FDG PET/CT imaging in patients with sepsis of unknown origin.

Triage score (n)	Positive FDG PET/CT findings, n (%)	Treatment planning (n)	Treatment modification (n); survived (n), expired (n)
0 (8)	2 (25)	No change (1)	
		Change (1)	D (1);
			survived (1)
1 (27)	17 (63)	No change (11)	
		Change (6)	D (2), S(4);
			survived (4), expired (2)
2 (18)	16 (89)	No change (10)	
		Change (6)	D (1), S (5);
			survived (5), expired (1)

S, surgery; D, drainage.

## Discussion

The identification of occult foci of infection in patients with sepsis of unknown origin can significantly reduce morbidity, relapse rates, and mortality through different mechanisms (prolongation of antibiotic therapy, switching from one antibiotic to another, drainage of an abscess, or surgical intervention) [11]–[13]. In the present study, FDG PET/CT successfully identified the foci of infection in 66% (35/53) of patients with sepsis of unknown origin. Notably, 13 (25%) patients had their treatment modified because of FDG PET/CT findings.

From a clinical standpoint, the various types of osteomyelitis may require different therapeutic strategies. A targeted and long-lasting antimicrobial therapy is of paramount importance in patients with acute hematogenous osteomyelitis. However, patients with persistent clinical signs may require the surgical removal of the sequestra and the resection of the infected bone and the surrounding soft tissue, especially in the presence of sepsis of unknown origin [14]. In this study, the majority of the study participants had musculoskeletal infections (38%, 19/53), especially in the spine. Twelve of these 19 patients had their treatment modified, and 9 of these subjects survived without evidence of disease. The results of the present study are in accordance with previous evidence indicating that PET is superior to both CT and MRI in the diagnosis of spine osteomyelitis, especially in patients with low-grade spondylitis [15], [16].

Several previous studies have tried to identify the predictors of positive FDG PET findings in patients with inflammatory and infectious diseases. Rabkin *et al.* [17] have shown that hyperglycemia or diabetes do not affect the rate of false-negative FDG PET results. Moreover, Vos *et al.* [18] demonstrated the clinical usefulness of FDG PET/CT for the detection of localized foci of infection in patients with severe neutropenia. The results from the present study indicate that liver cirrhosis was the only significant unfavorable predictor of positive FDG PET findings. This result may be explained by the changes in glucose metabolism occurring in cirrhotic patients. Granulocytes depend primarily on anaerobic glycolysis to supply the necessary energy for locomotion, chemotaxis, and phagocytosis. However, intracellular glycogen may serve as an alternative source of glycolytic substrate for phagocytosis in hypoglycotic, inflammatory exudates [19]. Patients with liver cirrhosis are characterized by an altered non-oxidative glucose disposal (glycogen storage) which reflects the impaired glycogen synthesis and a dysregulation in glucose-transporter expression [20], [21]. Because of the altered glycogen metabolism, granulocytes from patients with liver cirrhosis show deficient phagocytosis, ultimately resulting in a reduced uptake of FDG and a higher likelihood of negative PET findings.

The diagnosis of sepsis is based on physical examination, a battery of clinical measures, and various laboratory tests. In the present study, we have shown that normal AST and elevated Alk-P were significantly associated with the presence of positive FDG PET/CT findings in both univariate and multivariate analysis. Increased serum Alk-P levels may occur when liver space-occupying lesions or bone disorders are present [22]. In our study, 16 of the 19 patients with infections involving the musculoskeletal system had elevated Alk-P, and all of them showed positive FDG PET/CT findings. AST is a common surrogate marker of liver damage and its levels are significantly higher in patients with cirrhosis than in those with mild liver fibrosis [23]. Of the 14 study participants with a diagnosis of liver cirrhosis, 9 showed increased AST levels but only three had positive FDG PET/CT findings. In contrast, all of the 29 patients without liver cirrhosis had normal AST levels and only five of them had negative FDG PET/CT results.

A scoring system using the significant predictors of positive FDG PET/CT findings (normal AST and elevated Alk-P) was developed, which defined three distinct priority groups for FDG PET/CT imaging. There was a stepwise decrease in the probability of positive FDG PET/CT findings in patients with a score of 2, followed by those with a score of 1, until those who scored 0. This simple triage scoring system may serve as an initial guide to allocate patients with sepsis of unknown origin to FDG PET/CT imaging. However, the results of our study should be interpreted with caution because of its retrospective nature. More importantly, our scoring system needs external validation because our patients were selected for FDG PET/CT imaging at the discretion of the attending physician. Patients with complex medical conditions (e.g. end-stage renal disease or liver cirrhosis) were more likely to receive FDG PET/CT for diagnostic purposes. Therefore, we should give a caveat against overestimating the importance of liver disease in the proposed scoring system. Future well-designed and rigorously conducted prospective studies will be needed to determine how robust our priority assessment criteria were and the extent to which the patients selected were those who benefited the most from FDG PET/CT imaging.

To the best of our knowledge, the present study is the largest series thus far that reports on the clinical utility of FDG PET/CT in patients with sepsis of unknown origin. Our study yielded novel information with significant clinical implications. An important finding of the present report is that FDG PET/CT was able to identify occult foci of infection in 66% of the patients who had previously undergone an extensive diagnostic work-up with inconclusive data. Notably, a previous study conducted in our geographic area demonstrated that Ga-67 citrate scans were able to identify only 21% of such patients [24]. Interestingly, 25% of our study participants had their treatment modified after the localization of the infectious foci. The present study also demonstrates that liver cirrhosis is associated with a higher likelihood of negative FDG PET/CT findings, which may in part be explained by an impaired function of granulocytes during phagocytosis. In the interpretation of our findings, it should be noted that the prevalence rates of liver cirrhosis are substantially higher in Asian countries than in the United States. Most importantly, only 33% (3/9) of the patients with sepsis of unknown origin who had a diagnosis of liver cirrhosis accompanied by increased AST levels had positive FDG PET/CT findings. Another relevant finding of this study is the significant association between increased serum Alk-P levels and the higher likelihood of positive FDG PET/CT results, especially in patients with musculoskeletal infections. Finally, our data indicate that patients with normal serum AST and elevated Alk-P levels can take full advantage from FDG PET imaging.

Our study has some limitations. First, this is a single-center retrospective study and the results may not be generalizable to other populations. The spectrum of bacteria causing sepsis may vary in different countries and our study has been conducted in an area of endemic liver cirrhosis. Second, the lack of a variable gold standard makes the calculation of the accuracy of FDG PET/CT difficult. Third, our study may have a selection bias because FDG PET/CT was performed at the discretion of the attending physician. Therefore, the proposed scoring system should be independently validated in future studies. Finally, this study was not specifically designed to assess the clinical outcomes and the cost-effectiveness of FDG PET/CT in the management of patients with sepsis of unknown origin. Further research is needed to address these issues.

Pending external validation, our findings indicate that FDG PET/CT may be clinically useful for the detection of occult foci on infection in patients with sepsis of unknown origin.
